# At the crossroads of botanical collections and molecular genetics laboratory: a preliminary study of obtaining amplifiable DNA from moss herbarium material

**DOI:** 10.7717/peerj.9109

**Published:** 2020-05-26

**Authors:** Marta Saługa

**Affiliations:** Władysław Szafer Institute of Botany, Polish Academy of Sciences, Kraków, Poland

**Keywords:** CTAB DNA extraction, Herbarium, Maritime Antarctic, Mosses, Subantarctica, PCR, Plant, Sanger sequencing, Bryophytes

## Abstract

**Background:**

Research focused on extreme environments is often associated with difficulties in obtaining fresh plant material. Herbaria may provide great support as they house large collections of specimens from different parts of the world. Accordingly, there is also a growing interest in methods using herbarium specimens in molecular studies. Much of the literature on herbarium DNA is aimed to improve extraction and PCR amplification and is focused mostly on vascular plants. Here, I provide a brief study of DNA extraction efficiency from moss herbarium specimens, emphasizing the importance of herbaria as an invaluable source of material from hard-to-access geographical areas, such as the Antarctic region.

**Methods:**

The presented study is based on herbarium collections of 25 moss species collected in the austral polar regions between 1979 and 2013. The majority of samples were obtained using the DNeasy Plant Mini Kit (Qiagen, Hilden, Germany). The remaining, smaller part was extracted using an adapted CTAB-based approach. The performance of DNA extraction methods in terms of PCR amplification success was measured by testing several DNA fragments of various size. Furthermore, in order to estimate of DNA fragmentation level, an automated on-chip electrophoresis system was used.

**Results:**

Results reveal that DNA purity and the length of the target genetic region are the fundamental agents which drive the successful PCR reaction. Conversely, the DNA yield and specimen age seem to be less relevant. With this study, I present also an optimized CTAB-based approach which may effectively suppress inhibitors in the herbarium DNA. This method can be considered a cheaper alternative to column-based technology, particularly useful for dealing with a large number of samples. Results of this study confirmed previous reports and contribute to filling the existing gap in molecular analyses which involve the use of herbarium collections of mosses.

## Introduction

Exploration of a wealth of biological materials deposited in herbaria may have an invaluable potential for many types of researches for example, conservation, plant disease, plant invasion, environmental pollution, taxonomy, biogeography, and ecology (Lavoie, 2013). During recent years, an increasing number of studies have been focused on the use of herbarium specimens for molecular analyses (e.g., Staats et al., 2011; Särkinen et al., 2012; Do & Drábková, 2018; Höpke et al., 2019). However, the use of herbarium material in molecular studies is limited by the difficulty in obtaining amplifiable DNA. Extraction of high-quality genomic DNA from herbarium specimens is hindered mainly by its degradation into short fragments as well as chemical modifications which may result in failure of efficient genomic DNA isolation and/or subsequent PCR amplification (Staats et al., 2011). Damage in herbarium DNA is mostly related to the specimen-specific issues such as sample preparation method, with only a minor impact of subsequent preservation history, and specimen age (Särkinen et al., 2012). Moreover, the general success of DNA extraction and PCR amplification in plants are affected by group-specific factors, which include diversity of leaf texture, and content of organic compounds (Särkinen et al., 2012).

Despite this fact, for some taxonomic groups, focused reports of step-by-step improvements of herbarium DNA extraction and amplification methods are still scarce or lacking. This is the case of bryophytes, which contrasts with current interest in molecular-genetic studies in moss biology. Published moss phylogenies are based mostly on herbarium material. Bryological studies using Sanger sequencing are related predominantly to phylogenetic aspects, but without directly reporting on such technical aspects as specimen age, DNA purity and yield, and PCR amplification success. However, many of them are based on different DNA extraction techniques and chemistry used, and hence efficiency of the methods are expected to vary. Thus, sharing empirically tested modifications of molecular protocols for biological collections are of major importance (e.g., Lavoie, 2013).

Mosses, being organisms well adapted to many environmental constraints, are often key components of flora in inaccessible and ecologically inhospitable environments. One such example can be the Antarctic biome. Mosses are the main taxonomic group forming terrestrial plant cover here with altogether, 115 species known from Antarctica with their greatest diversity found in the maritime Antarctic (Ochyra, Lewis Smith & Bednarek-Ochyra, 2008; Sollman, 2015). Botanical exploration of the Antarctic continent and the maritime Antarctic islands is logistically highly restricted. Beheregaray (2008) reported that a very small proportion of all phylogeographic studies (0.6%) focused on the Antarctic continent. In particular, terrestrial plants from the Southern Hemisphere have been a special case of “missing the boat”. Meanwhile, a massive proportion of 88% of studies have been coming from the Northern Hemisphere (Beheregaray, 2008). Since then, there is a growing interest in research focused on the flora from the high-latitude ecosystems of the Southern Hemisphere. These studies are related mainly to biogeography, evolutionary history and ecology of the major cryptogamic groups such as mosses, liverworts, lichens and algae (e.g., Peat, Clarke & Convey, 2007; De Wever et al., 2009; Green et al., 2011, 2015; Fraser et al., 2014; Hebel et al., 2018). Moreover, the important role of Antarctica in plant phylogeography studies has recently been highlighted by Estrella et al. (2019).

In documenting and studying biogeographical patterns of the Antarctic flora without undertaking challenging and expensive field expeditions, the possibility of using herbarium collections play an indispensable role. Yet, this potential has been explored to a very limited extent so far. Lavoie (2013) reported 417 studies using herbarium specimens published from 1933 to 2012, among which only three articles concerned the Antarctic flora, primarily mosses, lichens and liverworts (Markham et al., 1990; Peat, Clarke & Convey, 2007; Ryan, Burne & Seppelt, 2009). However, these studies did not use molecular techniques. Lavoie (2013) underlined that only a small number of biogeographical and environmental studies (17) used herbarium specimens with molecular analyses. This is in contrast with the fact that DNA extraction and amplification protocols for plants from herbarium collections have been published since the early 2000s (Drábková, Kirschner & Vlĉek, 2002). Only recently have biogeographers and ecologists started to more widely explore moss herbarium specimens using molecular techniques. It was also reflected in taxonomy and phylogeography of Antarctic bryophytes, Clarke, Ayre & Robinson (2008), Wyber (2013), Pisa et al. (2014), Biersma et al. (2017), Rankin et al. (2017), Biersma et al. (2018a, 2018b), Câmara et al. (2019), De Freitas et al. (2018), Ronikier et al. (2018) and Saługa et al. (2018). Part of these works also used fresh samples from fieldwork in Antarctica. However, without a detailed examination of literature, it is difficult to determine if published moss phylogenies of Antarctic taxa using fresh specimens prevail over those using herbarium collections. Nevertheless, a rapid growth of molecular techniques coupled with exploration of Antarctic plant collections in herbaria worldwide may greatly stimulate documenting phylogeographical patterns in the Southern Hemisphere.

Currently, the largest bryophyte collections from Antarctica and Subantarctica are deposited in the herbaria of the British Antarctic Survey (BAS), Władysław Szafer Institute of Botany of the Polish Academy of Sciences (KRAM) and the Australian Antarctic Division (AAD) (now housed in the Tasmanian Herbarium in Hobart—HO). The most extensive and comprehensive description of all known species and varieties of moss in the Antarctic biome was performed by Ochyra, Lewis Smith & Bednarek-Ochyra (2008) in their “Illustrated moss flora of Antarctica” and the collections made by the author are deposited at herbarium KRAM. In South America, the largest collection of bryophytes from Antarctica is located at the University of Brasilia herbarium (UB), which is mostly associated with the Brazilian Antarctic program (PROANTAR) (Rosa et al., 2019).

In the course of a research project on the evolutionary history of the Antarctic bryophytes, conducted by the Institute of Botany, Polish Academy of Science in Kraków, including our last studies (Ronikier et al., 2018; Saługa et al., 2018), the feasibility of efficient use of herbarium collections was of particular importance. While the DNA extraction efficiency from Antarctic bryophyte species as such should not differ much from their counterparts from other regions (species specific traits), this may not always be the case for herbarium specimens (specimen specific traits). Here, the effect of specimen preparation method may be a decisive factor for DNA quality and subsequent molecular analyses. In this particular case, the sub-Antarctic and Antarctic environment is very challenging for researchers. Thus, to benefit from the potential DNA-friendly collection, the role of collector seems to be of crucial importance. It should be noted at this point that in the years when the examined collection (KRAM) had been harvested, future usage in molecular analyses was not considered. Until the current project, the unique moss collection from austral polar regions in KRAM has not been studied in molecular analyses. Thus, before starting an Antarctic project in our lab, assessing specifically whether moss specimens from sub-Antarctic and Antarctic areas, whose preparation was influenced by technically peculiar working conditions of remote and long-term field works, would yield sufficient amounts of DNA to secure the planned analyses, was of particular importance.

The present report contributes to filling the existing gap in the available molecular protocols using moss herbarium collections. As the first step of project, DNA extraction efficiency from moss herbarium material was tested with the aim of obtaining DNA isolates good enough for downstream Sanger DNA sequencing. To test this, I used samples of 25 moss species collected in the austral polar regions of the Maritime Antarctic and Subantarctic islands, between 1979 and 2013, as source for PCR amplification of selected target regions. Within this study, I tested several methods of DNA isolation including a modified CTAB-based DNA extraction protocol may be an alternative approach to the relatively more expensive column-based method.

The study is based on expert-verified sub-Antarctic and Antarctic moss specimens. Thus, in addition to the technical aspects, it provides DNA barcodes of previously inaccessible genetic information. It is important to emphasize the essential importance of a proper taxonomic determination of dry plant material. Taxonomic difficulties could be attributed, in particular, to bryophytes, due to their wide morphological variation and plasticity. In this study all plant material were collected and verified by Ryszard Ochyra (Institute of Botany, Polish Academy of Sciences), leading author of the “Illustrated moss flora of Antarctica” (Ochyra, Lewis Smith & Bednarek-Ochyra, 2008). DNA sequences from species and populations difficult to obtain are important for various applications (phylogeny, taxonomy, ecology, biodiversity, conservation). I would like therefore to underline the importance of herbaria as an invaluable source of material especially from hard-to-access regions.

## Materials and Methods

The present report of DNA extraction efficiency from moss herbarium material is divided into two parts. The first is based on a commercially available DNA extraction kit, DNeasy Plant Mini Kit (Qiagen, Hilden, Germany) and concerns the general feasibility of obtaining PCR-amplifiable DNA from moss herbarium specimens. Here, the following agents have been considered to amplification success: (1) DNA yield and purity; (2) age of herbarium specimens; (3) taxon studied; and (4) locus length. In addition, sequencing success has also been presented. The second part of this study involves a short comparison of CTAB based DNA extraction protocols, as well as a comparison of the CTAB methods with the DNeasy Plant Mini Kit. In this respect, the DNA extraction protocols with the best overall PCR success has been considered as the most appropriate for the analyzed material.

### Plant sampling

The herbarium specimens analyzed in this study are stored in the bryophyte herbarium of the Władysław Szafer Institute of Botany, Polish Academy of Sciences (KRAM). Altogether, the samples used for our tests represented 25 species from 11 families (Table S1). Plant material originated from a number of austral polar areas, namely: King George Island (South Shetland Islands) in the Antarctic, Marion Island (Prince Edward Islands), Île de la Possession (Îles Crozet) and Îles Kerguelen in the Subantarctic, and from Isla Grande de Tierra del Fuego (southern South America). They include also one specimen from Australia (Tasmania). Analysed specimens were in the age range from one to 35 years with a median of 15. As far as I could reconstruct, all specimens used in this study were air-dried after collection. There is no confirmation whether plant material was directly dried after harvesting or at the later stage. Neither chemical treatment, nor freezing regime were applied to the analyzed collections.

As part of the larger project, the study was based on collection of mosses from the polar region of the southern hemisphere (KRAM), which comprises unique specimens documenting the biodiversity of that region. Thanks to this I could assess the feasibility of DNA analyses on this specific material. However, there was a limited material available for DNA isolation to keep particular collections safe and it was not possible to replicate DNA extractions for individual samples. Finally, the two parts of the study were developed as independent experiments and therefore they are based on different sampling.

### Preparation steps

Sample preparation, DNA extraction and PCR-amplifications were carried out in the Laboratory of Molecular Analyses, Władysław Szafer Institute of Botany, Polish Academy of Sciences. No bryophyte samples had been investigated in the laboratory before the experiments reported here. The benchtop was cleaned with Fugaten Spray (Medilab, Poland) with 1-min incubation before all preparation steps. Forceps were sterilized with ethanol and flamed before each specimen handling. All disposable consumables were DNA-free. Sterile filter tips were used for all experimental procedures. During the preparatory step, whenever possible, green gametophyte vegetative shoots were taken. Considering that large amounts of herbarium voucher material are usually not available, I applied to the presented DNA extraction protocols less than 10 mg of dried tissue, typically around 8 mg. Selected fragments of the dried tissue from herbarium voucher specimens were weighted and disrupted in a mixer mill (MM400; TissueLyser II; Qiagen, Retsch, Germany), using one tungsten bead per sample. Samples were ground two times for 30 s at 20 Hz and subsequently used for DNA extraction.

### DNA extraction and DNA quality measurements

Total genomic DNA was extracted with four different protocols: column-based DNeasy Plant Mini Kit (Qiagen, Hilden, Germany), and three variants of CTAB-based extraction method. A detailed description and major variations of all three CTAB extraction methods used are summarized in Table 1. In the rest of this article, the DNA extraction methods are referred as Qiagen Kit, CTAB-ethanol/NaCl^a^, CTAB-ethanol/NaCl^b^, and CTAB-isopropanol, respectively. In the case of the Qiagen Kit, I followed the manufacturer’s instruction. CTAB-based protocols used emerged from the extraction methods described by Staats et al. (2011), Särkinen et al. (2012) and Healey et al. (2014), and vary according to the precipitation solution used.

**Table 1:** CTAB extraction test. DNA extraction protocol.

Add extraction buffer		
Add to the each sample 1 mL of preheated to 65 °C 2× CTAB buffer containing β-mercapthoethanol		
Preparation of 2× CTAB buffer (250 mL):		
25 mL (1 M) Tris-HCl pH 7.5 + 75 mL (5 M) NaCl + 12.5 mL (0.5 M) EDTA + 5 g CTAB + water until 250 mL		
Final concentration: (100 mm) Tris-HCl + (1.5 M) NaCl + (25 mm) EDTA + (2%) CTAB (w/v)		
Add immediately just before use: β-mercapthoethanol 0.3% (v/v): 5 μL/1,000 μl solution		
Incubate the sample at 65 °C for 60 min with mixing by inversion every 10 min		
Centrifuge at 5,000 rcf for 5 min to pellet and remove unlysed leaf tissue. Transfer the extract to a new 2 mL tubes		
Protein extraction and RNAse treatment		
Add an equal volume of chloroform: isoamyl alcohol (24:1) to the extract and mix gently. Extract for 30 min by rocking on orbital shaker		
Centrifuge at 13,000 rpm for 10 min		
Transfer the upper phase (containing DNA) to a new 2 mL tubes. Take care to avoid the aqueous/organic layer interface		
Add one μL of RNase A solution (10 mg/mL) per 100 μL DNA solution and incubate at 37 °C for 15 min with periodic, gentle mixing		
Repeat the chloroform: isoamyl alcohol extraction to clear the aqueous phase		
Precipitation		
Add *X* volume of 5 M NaCl to the transferred aqueous phase and mix gently by inversion. Then add *Y* volume(s) of pre-chilled (−20 °C) 95% ethanol and mix gently by inversion. Incubate at −20 °C for 60 min. Note: do not leave the sample at −20 °C for more than 60 min as both the CTAB and NaCl can precipitate from solution, preventing DNA isolation		Add 1.8 volume of pre-chilled (−20 °C) isopropanol to the transferred aqueous phase and mix gently by inversion. Incubate at −20 °C for 24 h
CTAB-ethanol/NaCl^a^	CTAB-ethanol/NaCl^b^	CTAB-isopropanol
*X* = 0.5 *Y* = 3 Healey et al. (2014)	*X* = 0.1 *Y* = 0.6 our modification	
	Attention	
	DNA pellets are poorly visible	
Centrifuge at 14,000 rpm for 20 min to collect precipitate, Pour off the liquid and add 750 μL of pre-chilled (−20 °C) 70% ethanol, Spin down DNA at 13,000 rpm for 15 min, Pour off the liquid and air-dry DNA pellet for 15–30 min at room temperature or dry the samples in vacuum centrifuge for 5 min. Note: in case of isopropanol precipitation wash the pellet 5 times with 750 μL pre-chilled (−20 °C) 70% ethanol		
Dissolve in Tris-EDTA buffer (TE buffer) pH 8.0		
Preparation of TE buffer (500 mL):		
5 mL (1 M) Tris pH 8 + 1 mL (0.5 M) EDTA pH 8 + water until 500 mL		
Resuspend DNA in 80 μL of TE buffer		

**Notes:**

a Mix the aqueous phase with 0, 5 vol. 5M NaCl and 3, 0 vol. 95% ethanol.

b Mix the aqueous phase with 0, 1 vol. 5M NaCl and 0, 6 vol. 95% ethanol.

DNA was eluted from the spin column (Qiagen Kit) with two successive elutions, each performed with 50 μL of elution buffer. The volume obtained after each centrifugation was pooled in one tube. In the case of the CTAB extraction test, the DNA pellet was resuspended directly in 80 μL of Tris-EDTA buffer. Extracted DNA was stored at −20 °C. The quality of the DNA extracts was estimated by running 3 μL of genomic DNA on a 1.0% agarose gel using Tris-borate-EDTA buffer and a Perfect™ 100 bp DNA ladder (Molecular Biology Products; EURx, Gdańsk, Poland). Gels were visualized under UV light after gel staining with SimplySafe™ (EURx, Molecular Biology Products, Poland). The concentration of DNA (ng·μL^−1^) extracted by Qiagen Kit and CTAB test was measured in all samples tested using Invitrogen Qubit™ 2.0 Fluorometer (Life Technologies, Eugene, OR, USA) with the Qubit™ dsDNA High Sensitivity Assay Kit. The Qubit™ working solution was made according to manufacturer’s instructions. I added 199 μL of working solution to each assay tube, and one μL of DNA to bring the final volume to 200 μL. A total of 10 μL of the Qubit™ DNA Standard solutions were used for standard tubes. Assay tubes were vortexed for 2–3 s, centrifuged briefly (~5 s), and then incubated at room temperature for 2 min to allow the assay to reach optimal fluorescence before measuring with the Qubit™ Fluorometer. Each tube was measured two times to test the reading accuracy.

### Qiagen extraction test

To verify the general feasibility of obtaining PCR-amplifiable DNA from moss herbarium specimens, Qiagen Kit was selected as a standard approach. This method was selected because of most of the recent bryological studies so far relied on this commercially available kit (e.g., Pisa et al., 2014; Wynns & Lange, 2014; Hedenäs, 2014, 2017; Biersma et al., 2017; Biersma et al., 2018a, 2018b). In this test, I analyzed 21 moss species of different age (8–35 years old). Isolation output was tested using PCR amplification of 10 genomic loci of variable length: nuclear ribosomal DNA (*5.8SR*-*ITS2*, *18S*, *adk* and *phy*2), and plastid marker regions (*psbAF*-*trnHR2*, *atpI*-*atpH*, *trnL*-*trnF*, *rps4, atpB1*-*rbcL1*, *psbB*-*clpP*). Genetic studies using herbarium specimens often highlight the degraded nature of ancient DNA. Hence, when the above PCR tests were negative, I additionally analyzed selected short fragments of the plastid *trnS*-*trnF* region.

### CTAB extraction test

The Qiagen Kit based isolation was compared with modified CTAB extraction protocols, less costly and potentially yielding a higher amount of isolated DNA. Here, I included different moss species which were collected over 1–15 years. To check the quality of the extracted genomic DNA, PCR amplification was performed for genetic regions of the nuclear ribosomal (*ITS5*bryo-*ITSC*bryo, *ITSD*bryo-*ITS4*bryo), and plastid (*trnT*-*trnF*, *rps4*) DNA regions. Within CTAB extraction protocols, the type of precipitation solutions, that is, ethanol combined with the sodium chloride, and isopropanol, as well as the proportions of the ethanol/sodium chloride used in relation to total sample volume, were the key determinants to test the effects on downstream molecular applications. I proposed a modified proportion of ethanol/sodium chloride component (here, protocol CTAB-ethanol/NaCl^b^), differing from the method used by Healey et al. (2014) (here, protocol CTAB-ethanol/NaCl^a^). The modification applied is supposed to increase DNA purity although possibly decreasing DNA concentration. Thus, I have checked whether DNA purity or concentration is more relevant for obtaining PCR-amplifiable DNA from herbarium moss tissue.

CTAB-based methods often provide a weakly purified DNA with contaminants having inhibitory effects on downstream enzymatic treatments, thus I attempted to additionally purify CTAB extracted samples. To this end, I used the Genomic DNA Clean & Concentrator-10 kit (Zymo Research, Irvine, CA, USA) according to the manufacturer’s recommendation. With this protocol, I used 10 μL of input genomic DNA. Following purification, DNA was eluted from the matrix with 15 μL of the DNA Elution Buffer preheated to 65 °C. It is worth noting that the Zymo-Spin matrix absorbs approximately five μL volume of the DNA Elution Buffer and the final output was around 10 μL of the purified genomic DNA. In all PCR reactions, I utilized two types of genomic DNA samples, before and after cleaning on the Zymo-Spin matrix, to compare PCR success rate between samples with and without purification. Although silica binding based protocols provide extractions of highly purified DNA samples, Qiagen Kit DNA isolates were also purified using Zymo-Spin matrix, to allow for a comparison of the final results within this assay.

In this test, to compare different extraction methods, the Agilent 2100 Bioanalyzer (Agilent Technologies, Waldbronn, Germany) an automated on-chip electrophoresis system, was used to evaluate the size distribution of the DNA fragments. The Agilent High Sensitivity DNA kit was used to provide the optimal separation of the potentially fragmented DNA. The samples were analyzed following the manufacturer’s protocol.

### PCR amplification and gel electrophoresis

Based on total DNA isolates, target regions were amplified using the primers listed in Table 2. The PCR of all plastid markers was carried out in accordance to the Shaw et al. (2007) “slow and cold” protocol, whereas nuclear markers were amplified due to the Sabovljević & Frahm (2011) recommendations. In all cases the total volume of PCR mixture was 20 μL and comprised of REDTaq DNA Polymerase (0.05 U/μL) (Sigma-Aldrich, St. Louis, MO, USA), 1× REDTaq Reaction Buffer containing MgCl_2_ (Sigma-Aldrich, St. Louis, MO, USA), primers forward and reverse (0.2 μm each primer) (Sigma-Aldrich, St. Louis, MO, USA), dNTPs solution (200 μm each dNTP) (Sigma-Aldrich, St. Louis, MO, USA), BSA (0.1 mg/mL) (New England BioLabs, Ipswich, MA, USA), one μL template DNA and water. The PCR products were run on agarose gel under the same conditions as DNA extracts (see above). All amplicon lengths included in the text are evaluated based on the mentioned gel electrophoresis (data not shown). I did not use any further improvement of selected PCR protocols, as long as my goal was to check the general feasibility of obtaining PCR-amplifiable DNA after Qiagen Kit and CTAB extractions.

**Table 2:** Genetic regions and sequence primers used for testing DNA quality through both Qiagen and CTAB extraction tests.

Region	Name	Sequence (5′–3′)	Target DNA region length (bp)	References
Nuclear genome markers				
*ITS*	*5,8SR*	TCGATGAAGAACGCAGCG	450	Hopple & Vilgalys (1999)
	*ITS2*	GCTGCGTTCTTCATCGATGC		White et al. (1990)
	*ITS5-*bryo	GGAAGGAGAAGTCGTAACAAGG	380	Sabovljević & Frahm (2011)
	*ITSC-*bryo	GCAATTCACACTACGTATCGC		Sabovljević & Frahm (2011)
	*ITSD-*bryo	CTCTCAGCAACGGATATCTTG	450	Sabovljević & Frahm (2011)
	*ITS4-*bryo	TCCTCCGCTTAGTGATATGC		Sabovljević & Frahm (2011)
*18S* rRNA	*NS1*	GTAGTCATATGCTTGTCTC	1,000	Cox et al. (2000)
	*PCRB*	TGATCCTTCCGCAGGTT		Cox et al. (2000)
*adk* gene	*adk* forward	GAAGAAGCCAGAAAACTGGGC	1,000	McDaniel & Shaw (2005)
	*adk* reverse	GTCACCCCATCTTCAGCAAC		McDaniel & Shaw (2005)
*phy2* gene	*phy2* forward	GGCATGGAAATGATGTGTTG	1,000	McDaniel & Shaw (2005)
	*phy2* reverse	CATCACTGTACCCATCTCG		McDaniel & Shaw (2005)
Plastid genome markers				
*psbAF*-*trnHR2*	*psbAF*	GTTATGCATGAACGTAATGCTC	250	Stech & Frey (2008)
	*trnHR-2*	CGCGCATGGTGGATTCACAATCC		Stech & Frey (2008)
*atpI*-*atpH*	*atpI*	TATTTACAAGYGGTATTCAAGCT	550	Shaw et al. (2007)
	*atpH*	CCAAYCCAGCAGCAATAAC		Shaw et al. (2007)
*trnL*-*trnF*	*trnL* (UAA) 5′Exon	CGAAATTGGTAGACGCTGCG	450	Quandt & Stech (2004) (primer C)
	*trnF* (GAA)	ATTTGAACTGGTGACACGAG		Quandt & Stech (2004) (primer F)
*trnT-trnF*	*trnT* (UGU)	CATTACAAGTGCGACGCTCT	1,500	Quandt & Stech (2004) (primer A)
	*trnF* (GAA)	see above		Quandt & Stech (2004) (primer F)
*rps5’-trnS*	*rps5′*	ATGTCCCGTTATCGAGGACCT	650	Souza-Chies et al. (1997)
	*trnS*	TACCGAGGGTTCGAATC		Souza-Chies et al. (1997)
*atpB1*-*rbcL1*	*atpB1*	ACATCKARTACKGGACCAATAA	650	Chiang, Schaal & Peng (1998)
	*rbcL1*	AACACCAGCTTTRAATCCAA		Chiang, Schaal & Peng (1998)
*psbB*-*clpP*	*Bry_psbB*	ATGAACACGATACCTAGGYAAACC	1,000	Piñeiro et al. (2012)
	*Bry_clpP1,2*	CATTGAAGCAGCTAATCCC		Piñeiro et al. (2012)
Selected fragments of *trnS*-*trnF* region				
*rpsM’-trnS*	*rpsM’*	TAGACATATTTTAGTTAATGG	500	Souza-Chies et al. (1997)
	*trnS*	see above		Souza-Chies et al. (1997)
*rpsM’-rps3′*	*rpsM’*	see above	250	Souza-Chies et al. (1997)
	*rps3′*	ATATTCTACAACTAACAACTC		Souza-Chies et al. (1997)
*rpsM2-rpsM*	*rpsM2*	TTTTACTACAACTACTTGAGA	100	Souza-Chies et al. (1997)
	*rpsM*	CCATTAACTAAAATATGTGT		Souza-Chies et al. (1997)
*rpsM1-rpsM*	*rpsM1*	CAATATCGTATTCGTCTAGAA	200	Souza-Chies et al. (1997)
	*rpsM*	see above		Souza-Chies et al. (1997)
*rps5′-rpsM*	*rps5′*	see above	300	Souza-Chies et al. (1997)
	*rpsM*	see above		Souza-Chies et al. (1997)
*trnT-trnL*	*trnT* (UGU)	see above	400	Quandt & Stech (2004) (primer A)
	*trnL* (UAA) 5′Exon	TCTACCAATTTCGCCATACC		Quandt & Stech (2004) (primer B)
*trnL* intron	*trnL* (UAA) 5′Exon	see above	500	Quandt & Stech (2004) (primer C)
	*trnL* (UAA) 3′Exon	GGGGGTAGAGGGACTTGAAC		Quandt & Stech (2004) (primer D)
*trnL-trnF*	*trnL* (UAA) 3′Exon	GGTTCAAGTCCCTCTATCCC	200	Quandt & Stech (2004) (primer E)
	*trnF* (GAA)	see above		Quandt & Stech (2004) (primer F)
*trnL* intron	trnL (UAA) 5′Exon	see above	200	Quandt & Stech (2004) (primer C)
	trnL (UAA) intron	GTTTCCTTTGAGTCTCTGCAC		Quandt & Stech (2004) (primer D_x_)
*trnL* intron	trnL (UAA) 5′Exon	see above	200	Quandt & Stech (2004) (primer C)
	trnL (UAA) intron	CTTCCATTGAGTCTCTGCACC		Quandt & Stech (2004) (primer D_i_)
*P6* loop	*P6* loop-g	GGGCAATCCTGAGCCAA	100	Taberlet et al. (2007)
	*P6* loop-h	CCATTGAGTCTCTGCACCTATC		Taberlet et al. (2007)

### Sequence analysis

Successful amplification products from Qiagen Kit extraction test were treated using enzymatic purification with ExoSAP-IT kit (Affymetrix, Santa Clara, CA, USA). I mixed three μL of template DNA with one μL of ExoSAP-IT solution, and incubated this mixture at 37 °C for 15 min, and at 80 °C for subsequent 15 min. Cycle sequencing reactions (3 min 96 °C, 30 cycles (10 s 96 °C, 5 s 50 °C, 2 min 60 °C)) were carried out in an Mastercycler Nexus thermocyclers (Eppendorf, Hamburg, Germany) using the BigDye Terminator 3.1 chemistry (Thermo Fisher Scientific, Waltham, MA, USA) along with the BDX64 Enhancing Buffer (MCLAB, San Francisco, CA, USA) in accordance with the manufacturer’s protocol for 32× dilution. However, in this case, several duplicate samples were taken to test more fold dilutions, here 64× and 128×. To sum up, all dilutions tested resulted in high-quality data with no significant differences between the obtained sequences. Cycle sequencing reactions were conducted using primers used for PCR amplification. Sequencing reactions were separated in the Applied Biosystems 3130 Series Genetic Analyzer (Thermo Fisher Scientific, Waltham, MA, USA). The sequence dataset was aligned with Geneious v.10.1.3 (Biomatters Ltd., Auckland, New Zealand), using default settings. GenBank accession numbers are listed in Table S2. All DNA sequences generated with this study may be downloaded directly by Data S1.

### Statistical analyses

Statistical analyses were performed to test for significance of differences observed among categories of samples. Calculations related to conducted statistical analyses are presented in File S1. Specific algorithm steps are based mainly on the linear regression analysis, and Kolomogorov–Smirnov two-sample test (hereafter, K–S) (Young, 1977; Zielinski & Zielinski, 1990). The nonparametric K–S test has been chosen, as is used for the objective statistical analysis of histogram data with no prior knowledge about sample distribution. Accordingly, within our data, it was a possibility to differentiate one complete histogram from another based on median values. Here, *D*-statistic is compared against *D* critical value of given confidence level and the number of samples utilized in the study. The K–S test has been supplemented by the linear regression analysis. Estimation of the *F*-statistic permits to identify whether examined variables have a greater influence on the PCR success or not. The K–S test outcomes have been compared against the corresponding linear regression analysis.

## Results

### Qiagen kit extraction test

**Figure 1:**
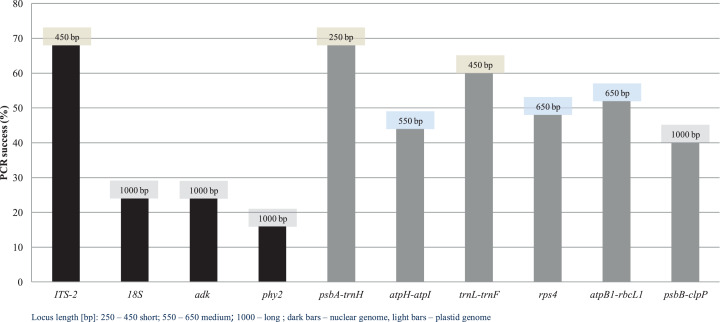
Qiagen extraction test. PCR success (%) of selected genetic regions used, measured as the number of positive amplicons divided by the total number of samples.

This test included 25 specimens that represented 21 moss species. The PCR and sequencing success for the individual herbarium specimen, and the 10 selected target regions are summarized in Table 3 and Fig. 1.

**Table 3:** Qiagen extraction test. Specimen information and PCR amplification success. Sequences lengths are estimated with the Geneious software after removal low-quality ends.

No.	Species	Coll. #	Origin^1^	Age^2^	gDNA (ng/μL)	*5.8 sr ITS2*	*18S*	*adk gene*	*phy2 gene*	*psbAF trnHR2*	*atpI atpH*	*trnL trnF*	*rps4 gene*	*atpB1 rbcL1*	*psbB clpP*
Colored background (successfull PCR/sequence length (bp)*)															
1.	*Andreaea depressinervis* Cardot	4928/79	KGI	35	0.466	no data									
2.	*A. nitida* Hook.f. & Wilson	3745/06	KER	08	4.060										
3.	*Blindia magellanica* Shimp.	611/99	MAR	15	1.920	458				246	374	468	658	602	
4.	*Brachythecium subplicatum* (Hampe) A.Jaeger	614/99	MAR	15	3.400	439	782*		no data	256	557	443	648	680	1,097
5.	*Breutelia integrifolia* (Taylor) A.Jaeger	124/06	POSS	08	0.890	448		no data		247	469	468	660	653	637*
6.	*Bucklandiella heterostichoides* (Cardot) Bednarek-Ochyra & Ochyra	3804/06	KER	08	1.630	443				244		316*			
7.	*Cratoneuropsis chilensis* (Lorentz) Ochyra	403/99	MAR	15	1.310	389				256	557	420	676	689	676*
8.	*Distichium capillaceum* (Hedw.) Bruch & Schimp.	1198/06	KER	08	1.430	449		no data	714	228	582	511	665	659	1,045
9.	*Ditrichum strictum* (Hook.f. & Wilson) Hampe	194/06	POSS	08	0.396	471	754*		895	244	499	169	665	629	449*
10.	*Hymenoloma antarcticum* (Müll. Hal.) Ochyra	2662/80	KGI	34	1.720	no data				246					
11.	—	Komárek s.n.	KGI	25	0.594					251		501		573	
12.	*Hymenoloma tortifolium* (Hook.f. & Wilson) Ochyra	3344/06	KER	08	8.780										
13.	*Notoligotrichum trichodon* (Hook.f. & Wilson) G.L.Sm.	487/95	FUE	19	too low										
14.	*Polytrichadelphus magellanicus* (Hedw.) Mitt.	302/95	FUE	19	too low										
15.	*Racomitrium lanuginosum* (Hedw.) Brid.	15/06	POSS	08	3.340	no data	no data	no data		238	528	470	660	671	547*
16.	*Sanionia uncinata* (Hedw.) Loeske	2268/80	KGI	34	4.000										
17.	—	2/06	POSS	08	1.710	437	no data			247	563	418	609	676	1,078
18.	*S. georgicouncinata* (Müll. Hal.) Ochyra & Hedenäs	454/80	KGI	34	3.800	433	774*	693*		255	540	434	655	632	643*
19.	*Schistidium falcatum* (Hook.f. & Wilson) B. Bremer	437/80	KGI	34	2.180	no data		no data		246			646	no data	
20.	—	408/95	FUE	19	0.256	394				248		no data			
21.	—	1447/99	MAR	15	1.810	no data				238	423*	434			
22.	*S. halinae* Ochyra	2711/80	KGI	34	0.722	396				243		434	no data	450	no data
23.	*Schistidium species* Ochyra	2022/06	KER	08	4.780										
24.	*Valdonia microcarpa* (Mitt.) Ochyra	555/99	MAR	15	1.310										
25.	*Warnstorfia fontinaliopsis* (Müll. Hal.) Ochyra	1193/80	KGI	34	1.120	431	777*	no data	no data	251	552	417	665	624	548*

**Notes:**

* Single-stranded read.

1 More collection site details are given in Table 1.

2 Age of the specimen in the year of DNA extraction.

no data, unsuccessful sequencing.

FUE, Isla Grande de Tierra del Fuego, southern South America; KER, Îles Kerguelen; KGI, King George Island, South Shetland Islands; MAR, Marion Island, Prince Edward Islands; POSS, Île de la Possesion, Îles Crozet.

### Effect of the DNA yield from herbarium specimens

Statistically, there is no significant effect of DNA concentration level on the PCR success, as has been revealed by the K–S test and the linear regression analysis. Both have been estimated at the 95% confidence level (File S1). In this study, the Qiagen Kit extraction method generally yielded low amounts of total DNA with concentration ranging from 0.256 to 8.780 ng/μL with two samples below detection level (Table 3) and (File S2). A vast majority of samples (48%) yielded between 0 and 1.500 ng/μL. However, obtained low yield of DNA template from herbarium samples seems not to be a limiting factor for the successful PCR amplification. To illustrate, number of specimens with one of the lowest amount of genomic DNA amplified successfully in more than five regions tested that is, *Ditrichum strictum* (0.396 ng/μL; 9 genetic regions), *Schistidium halinae* (0.722 ng/μL; 6 genetic regions), and *Breutelia integrifolia* (0.890 ng/μL; 8 genetic regions). Interestingly, specimens with the highest genomic DNA concentration failed to amplify in all target region tested that is, *Andreaea nitida* (4.060 ng/μL), *Schistidium species* (4.78 ng/μL), and *Hymenoloma tortifolium* (8.78 ng/μL). No amplicon was obtained in two samples where no genomic DNA was detected (i.e., *Notoligotrichum trichodon*, *Polytrichadelphus magellanicus*). With regard to agarose gel electrophoresis, the DNA extracts were not visible under UV light.

### Age of herbarium specimens and PCR success

The K–S test has shown that the age of the sample, in the range tested in the study, does not affect the PCR reaction. Conversely, applied linear regression model has shown the correlation between examined variables. Both tests have been performed at the 95% confidence level (File S1). To illustrate, several of the youngest specimens (i.e., 8 years old, *Distichium capillaceum*, *Ditrichum strictum*, and *Racomitrium lanuginosum*) along with the oldest (i.e., 34 years old, *Sanionia georgicouncinata*, *Schistidium halinae*, and *Warnstorfia fontinaliopsis)* amplified successfully in more than five regions tested, including both nuclear and chloroplast regions. On the other hand, certain of analyzed specimens, both from the oldest (i.e., 34 and 35 years old, *Sanionia uncinata*, and *Andreaea depressinervis*), and the youngest groups (i.e., 8 years old, *Andreaea nitida*, *Hymenoloma tortifolium*, and *Schistidium* sp.) collections did not amplify in any or at most at one of the selected genetic regions.

### Locus length and the PCR and sequencing success

A total of 80 sequences (product sequencing in two directions) were obtained from an assay of 10 DNA loci. Three of the target regions were the most difficult to amplify, with PCR success rates in the range of 16–24% for the nuclear *18S*, *adk*, *phy2* genes. These markers are the longest target regions selected for this study (all region length ca. 1,000 bp). On the other hand, PCR success for the longest chloroplast region (here, 1,000 bp *psbB*-*clpP*) was not as low as for the above nuclear amplicons.

In the order of PCR success, the following genetic regions can be specified: *phy2* (16%; ca. 1,000 bp), *adk* and *18S* (24%; ca. 1,000 bp), *psbB*-*clpP* (40%; ca. 1,000 bp), *atpI*-*atpH* (44%; ca. 550 bp), *rps4* (48%; ca. 650 bp), *atpB1*-*rbcL1* (52%; ca. 650 bp), *trnL*-*trnF* (60%; ca. 450 bp), *psbAF*-*trnHR2* (68%; ca. 250 bp), and *5.8SR-ITS2* (68%; ca. 450 bp) (Fig. 1). With regard to success of bidirectional product sequencing from obtained amplicons, it amounts to: 0% for *18S* and *adk* gene, 30% for *psbB*-*clpP*, 67% for *phy2* gene, 71% for *5.8SR-ITS2*, 87% for *trnL*-*trnF*, 91% for *atpI*-*atpH*, 92% for *rps4* gene and *atpB1*-*rbcL1*, and 100% for *psbAF*-*trnHR2*. The quality of DNA sequences was mostly high and ranged from ca. 250 bp for *psbAF*-*trnHR2* to ca. 1,000 bp for *psbB*-*clpP* regions. The good quality single-strand data were also obtained in a few cases, and they are marked in Table 3 with an asterisk. Despite short fragments were targeted, using selected fragments of *trnS*-*trnF* region samples that failed into PCR reaction previously (i.e., *Andreaea depressinervis*, *A. nitida*, *Sanionia uncinata*, *Schistidium* sp.) still did not amplify.

### CTAB extraction test

**Figure 2:**
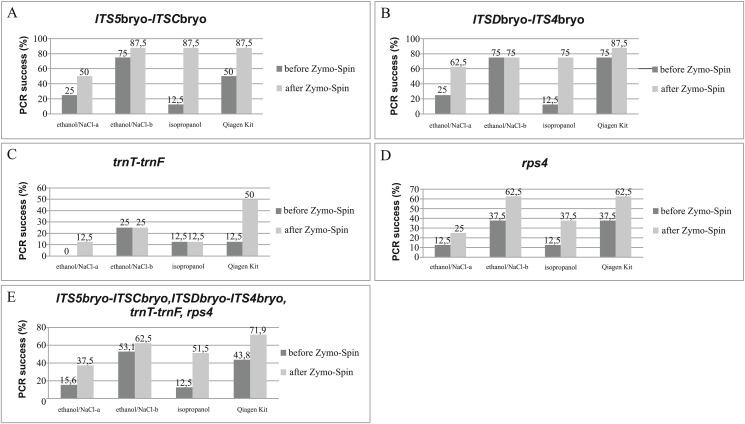
CTAB extraction test. Effect of extraction method on PCR success (%) measured as the number of positive amplicons divided by the total number of samples, before and after using Genomic DNA Clean & Concentrator-10 kit. (A) *ITS5*bryo-*ITSC*bryo, (B) *ITS5D*bryo-*ITS4*bryo, (C) *trnT-trnF*, (D) *rps4*, (E) *ITS5*bryo-*ITSC*bryo, *ITS5D*bryo-*ITS4*bryo, *trnT-trnF*, *rps4*.

The efficiency of DNA extraction using CTAB-ethanol/NaCl^a^, CTAB-ethanol/NaCl^b^, CTAB-isopropanol, and Qiagen Kit procedures are compared according to PCR success (Fig. 2). Four DNA fragments (nuclear *ITS5*bryo-*ITSC*bryo, *ITSD*bryo-*ITS4*bryo, and plastid *trnT*-*trnF*, *rps4*), were amplified before and after additional purification using Zymo-Spin matrix. The comparison of four extraction methods showed differences in the number of successfully amplified target regions. The extraction methods of CTAB-ethanol/NaCl^b^ with a modified proportion of ethanol/NaCl components and Qiagen Kit have the best overall PCR success. However, when compared CTAB-ethanol/NaCl^b^, and Qiagen Kit extraction before additional purification, the method that yielded the most amplifiable DNA is CTAB-ethanol/NaCl^b^, whereas, after purification treatment, the best performance showed Qiagen Kit extraction. Remaining tested protocols, CTAB-ethanol/NaCl^a^, and CTAB-isopropanol have significantly worse performance, in particular before DNA cleaning. To summarize, additional purification and concentration using Zymo-spin matrix significantly improved the PCR output in all DNA extraction methods tested. The highest PCR success increase was reported in the CTAB-isopropanol method (an increase of 39%). For the remaining DNA extractions, the success of PCR amplification after Zymo-spin matrix purification was increased by 28.1% for the Qiagen Kit, 21.9% for the CTAB-ethanol/NaCl^a^, and 9.4% for the CTAB-ethanol/NaCl^b^ procedure. Furthermore, an improvement of the genomic DNA concentration was observed after using Zymo-Spin kit through all extraction methods tested (Table 4).

**Table 4:** CTAB extraction test. Specimen information and DNA yield measured before and after using Genomic DNA Zymo Clean & 3Concentrator-10 kit.

No.	Species	Coll. #	Origin^1^	Age^2^	gDNA (ng/μL)							
					Before Zymo-Spin				After Zymo-Spin			
					CTAB-ethanol/NaCl^a^	CTAB-ethanol/NaCl^b^	CTAB-iso.	Qiagen Kit	CTAB-ethanol/NaCl^a^	CTAB-ethanol/NaCl^b^	CTAB-iso.	Qiagen Kit
1.	*Brachythecium rutabulum* (Hedw.) Shimp.	1363/99	MAR	15	2.120	1.920	2.080	2.410	10.800	6.850	10.225	8.112
2.	*Breutelia integrifolia* (Taylor) A.Jaeger	3597/06	KER	08	0.920	0.124	1.170	0.314	5.250	2.370	8.550	2.150
3.	*Bucklandiella striatipila* (Cardot) Bednarek-Ochyra & Ochyra	3758/06	KER	08	0.845	0.025	0.444	0.030	2.500	1.650	2.750	1.956
4.	*Cratoneuropsis chilensis* (Lorentz) Ochyra	403/99	MAR	15	6.985	3.120	6.720	1.640	15.125	10.000	17.120	7.125
5.	*Cratoneuropsis chilensis* (Lorentz) Ochyra	1448/99	MAR	15	4.920	4.480	17.000	5.160	15.025	15.500	51.336	22.650
6.	*Holodontium strictum* (Hook.f. & Wilson) Ochyra	3581/06	KER	18	1.680	0.748	2.550	0.700	6.780	3.250	9.656	2.850
7.	*Rhacocarpus purpurascens* (Brid.) Paris	613/13	TAS	01	0.656	0.540	1.510	0.256	2.050	2.450	4.885	2.100
8.	*Valdonia microcarpa* (Mitt.) Ochyra	555/99	MAR	15	1.190	1.170	2.660	1.020	4.450	4.200	8.750	3.850

**Notes:**

1 More collection site details are given in Table 1.

2 Age of the specimen in the year of DNA extraction.

a Mix the aqueous phase with 0, 5 vol. 5M NaCl and 3, 0 vol. 95% ethanol.

b Mix the aqueous phase with 0, 1 vol. 5M NaCl and 0, 6 vol. 95% ethanol.

KER, Îles Kerguelen; MAR, Marion Island, Prince Edward Islands; TAS, Australia, Tasmania.

The length of the target region appeared to strongly influence the amplification success. Accordingly, the *trnT*-*trnF* locus was the longest (ca.1,500 bp), and the most difficult to amplify. Nevertheless, the Qiagen Kit extraction method in combination with Genomic DNA Clean & Concetrator-10 kit has proved to be the most effective for the successful amplification of the above-mentioned genetic region. Remaining loci (*rps4* ca. 600 bp; *ITSD*bryo-*ITS4*bryo ca. 450 bp; *ITS5*bryo-*ITSC*bryo ca. 380 bp) were comparable with respect to PCR success with a small advantage for nuclear *ITS* regions.

The electropherograms obtained by automatic fragment sizing within all extraction methods showed a broad distribution of bands which has indicated that genomic DNA is highly fragmented (Fig. S1). The average sizing of DNA isolate did not vary significantly across methods and is ranged from ca. 400 to 500 bp. Despite the fragment size distribution in all electropherograms remains comparable, the most similar shape of the genomic DNA profiles can be observed among CTAB-ethanol/NaCl^b^, and Qiagen Kit extraction method, which could be also reflected in comparable PCR success rate within these two assays. Similarly to the Qiagen Kit extraction test, DNA extracts were not visible under UV light after agarose gel electrophoresis.

## Discussion

### DNA purity rather than concentration as a key factor

Presented comparisons highlight the key importance of DNA purity after isolation from the herbarium sample, rather than DNA quantity, for successful PCR. The differences between various DNA concentration levels and PCR success turned out to be not statistically significant. Thus, particular attention should be paid to separating DNA from naturally occurring plant cell contaminants, rather than strenuous efforts to obtain high DNA quantity. Among protocols tested in my study, the CTAB-based DNA extraction method provides such a solution, making it a superior choice relative to silica gel column-based commercial kits for DNA extraction.

In the CTAB extraction protocols applied, the performance of the ethanol/NaCl solution proved to be crucial for obtaining pure DNA. More precisely, decreasing the volume of the ethanol/NaCl solution to the total volume of extracted sample, that is, CTAB-ethanol/NaCl^b^ protocol, caused a significant increase of PCR success as opposed to the original proportions applied by Healey et al. (2014). In turn, the proportion of the ethanol/NaCl ingredients has remained unchanged in both variants of the CTAB-ethanol/NaCl based protocols. Likely, reducing the volume of ethanol in the original CTAB protocol may have resulted in a reduced amount of precipitated genomic DNA but in the same time in a significantly lowered concentration of the co-precipitated PCR inhibitors, such as polysaccharides, phenols, and other organic compounds. In general, the addition of a high salt buffer (here, NaCl) could increase genomic DNA purity by a boost of polysaccharides solubility in ethanol, allowing their removal when DNA is pelleted under centrifugation step.

In the CTAB-ethanol/NaCl^b^ extraction protocol, the measured concentration values were the lowest across the tested protocols. It is assumed that DNA yield is good enough to obtain acceptable PCR products if ranged between 6.0 and 100 ng/μL (Do & Drábková, 2018). In my study, I obtained successful PCR reactions from samples with concentration values lower than one ng/μL. Nevertheless, the best performing DNA protocols should be aimed to obtain high purity combined with high DNA yield, which is particularly important in respect of high-throughput sequencing methods.

In CTAB extraction tests, the Genomic DNA Clean & Concentrator-10 kit was additionally applied to all prepared DNA extracts. This kit is expected to provide ultra-pure, high-yield genomic DNA. Accordingly, DNA concentration and percentage of the successfully amplified samples has risen significantly after Zymo-Spin cleaning. The main increase in PCR success was observed in the case of the potentially most contaminated extracts, which derived in this study from CTAB-isopropanol, and CTAB-ethanol/NaCl^a^ protocols.

Presented results are congruent with several studies which concluded that DNA purity is more important for amplification success than DNA yield (e.g., Höss & Pääbo, 1993; Hänni et al., 1995; Kalmár et al., 2000; Rohland & Hofreiter, 2007; Särkinen et al., 2012). It is worth emphasizing, that plant material could be especially prone to PCR inhibition compared to other organisms. The presence of primary and secondary chemicals in plant cells are expected to have inhibiting properties on PCR reaction. Several different chemical constituents have been found in bryophytes so far (Klavina et al., 2012, 2015). As an example, Sabovljević, Bijelović & Dragoljub (2001) described bryophytes as “remarkable reservoir” of natural products and/or secondary compounds such as terpenoids, phenols, glycosides, fatty acids and rare aromatic ingredients. This is also confirmed by Soni & Kumar (2009) who underlined that extraction of DNA from bryophytes could be very difficult due to the presence of secondary compounds inhibiting downstream applications. The range of compounds in bryophytes is dependent mostly on species. Nevertheless, the impact of environmental conditions could be equally important. Importantly, within one species, the range of chemistry variation is lower as qualitative compared to quantitative contents. The season of the collection as well as the treatments of the material after the collection time prove to be also substantial (M. Sabovljević, 2019, personal communication).

It cannot be ruled out that particular species, containing their distinctive chemical compounds, present intrinsic problems for successful DNA amplification. Here, one sample per species was used as the Subantarctic moss collections which were a focus of this study, due to limitation of accessible material. Thus, more data would be needed to draw a conclusion about whether and to what extent obtained results may be correlated with specific taxa concerned.

### Effects of target amplicon size and specimen age on successful PCR

In the tests of extraction protocols, length of the selected target regions was correlated with the PCR amplification success. This appears an obvious tendency for highly degraded genomic material and presented results are in agreement with Särkinen et al. (2012) and Do & Drábková (2018) who indicated that the most easily amplifiable DNA fragments from herbarium material are those below 500 bp. In this report, based on Qiagen Kit extraction test, the best-performing locus are *psbAF*-*trnHR2* (ca. 250 bp), *trnL*-*trnF* (ca. 450 bp), and *5.8SR*-*ITS2* (ca. 450 bp). The most pronounced decrease in PCR success was observed in amplicons around 1,000 bp (*18S*, *adk*, *phy2*, *psbB*-*clpP*, including *trnT*-*trnF* region from CTAB extraction test), and was more evident in nuclear regions. However, it was possible in some cases to amplify target genomic regions of up to 1,500 bp. Since short fragments prevail in herbarium DNA, it is expected that PCR of smaller regions has a higher success rate. On the other hand, an attempt to amplify short, barcode regions using samples which failed previously in PCR reaction (within Qiagen Kit extraction test) was still unsuccessful. In a case like this, DNA un-purity may play a more significant role in inhibiting PCR reaction than DNA fragmentation. Possibly, in this particular case, PCR optimization, using both fresh and herbarium material may result in improvement of successful amplification.

The specimen age effect on amplification was not clearly resolved in the present analyses. The K–S statistical test has shown that the age of the sample does not affect PCR reaction, whereas applied linear regression model has shown the correlation between examined variables. However, it should be pointed out that the number of the individuals in a given age was unequal, which could have influenced the obtained results. Successful amplification rate was comparable for the oldest (34 years old) and youngest specimens (8 years old) and rather other factors like the collection history, DNA purity (taxon studied), and locus length seem to be decisive. Previous studies have also shown that age of herbarium samples had no significant effect on PCR success, pointing out the importance of locus types to be amplified rather than the age (Särkinen et al., 2012; Do & Drábková, 2018). Summarizing, the age of moss specimen should not deter bryologists from their usage in molecular research although certainly at the sample age much exceeding those tested here the impact of DNA fragmentation may gradually appear preponderant.

### General DNA degradation in moss herbarium material—CTAB test

In this study the level of DNA fragmentation in moss herbarium samples extracted by four different protocols was also considered. The overall strand breaks of DNA retrieved from the selected moss herbarium specimens was high and only slightly varied between applied extraction methods, and specimens. However, based on obtained electropherograms it is possible to notice that the quality of genomic DNA was the most similar for the Qiagen and CTAB-ethanol/NaCl^b^ extraction methods. Likely, the comparable level of PCR success obtained based on these two methods can be largely attributed to this and suggests that the modified CTAB-based protocol could offer high-quality DNA from herbarium moss collections, which could correspond to results obtained with Qiagen protocol.

I also found no ample difference between obtained DNA profiles for all samples tested, representing age range between 1 and 15 years. Although Weiß et al. (2016) documented the correlation of DNA degradation through time, our samples did not show any age-related fragmentation in the time frame tested. On the other hand, it has been suggested that most DNA fragmentation in herbarium samples occurs on specimen preparation by applying sample drying using a high temperature (60 °C) or alcohol (Staats et al., 2011; Särkinen et al., 2012). Consequently, in our tests, most DNA damage could likely be attributed to sampling method preparation, here air-drying applied after collection although the number of specimens used in this test is too small to draw firm conclusions.

Certainly, in any case, it is important to underline the need for collecting and gathering DNA-friendly material accompanying herbarium collections during expeditions. This could be mostly obtained by using immediate silica gel drying, using appropriate collection buffers or Whatman FTA card technology, as emphasized by Gaudeul & Rouhan (2013).

## Conclusions

This report is the first to offer a ready-to-use CTAB-based DNA extraction protocol tested specifically for moss herbarium specimens. This procedure provides a good alternative to expensive commercial kits, without negatively influencing experiment success. According to presented tests, the quality and quantity of DNA obtained with this method is high enough for downstream PCR and Sanger DNA sequencing. Observations regarding factors which influence the usage of moss herbarium material for DNA isolation are congruent with previous studies based on other groups of organisms. DNA purity and targeted amplicon size are more correlated with PCR success than DNA yield. It is also showed that examined genomic DNA was highly fragmented, as typical for collection material, but degradation was not correlated with collection age. Methodological conclusions could be directly adaptable to various molecular studies on mosses based on herbarium material. This seems of special value when taking into account that mosses are main elements of flora in many geographical areas difficult to reach due to field work logistics constraints. Antarctica and the austral polar region, in general, can serve as the prominent example. In such cases, the possibility to efficiently include herbarium specimens in investigation appears of key importance.

## Supplemental Information

10.7717/peerj.9109/supp-1 Supplemental Information 1 List of species tested with a detailed description of the collection site.

10.7717/peerj.9109/supp-2 Supplemental Information 2 Qiagen extraction test. Genbank accession numbers for the studied specimens.

10.7717/peerj.9109/supp-3 Supplemental Information 3 CTAB extraction test. Electropherograms of genomic DNA analyzed using the Agilent High Sensitivity DNA kit on the Agilent 2100 Bioanalyzer System. Abbreviation used: [bp] base pairs; [FU]-fluorescence units (DNA amount); [s]-seconds.

10.7717/peerj.9109/supp-4 Supplemental Information 4 Qiagen test. Statistical analyses.

10.7717/peerj.9109/supp-5 Supplemental Information 5 Qiagen test. DNA concentration intervals.

10.7717/peerj.9109/supp-6 Supplemental Information 6 DNA sequences generated with this study.

## Additional Information and Declarations

### Data Availability

The following information was supplied regarding data availability: All plant material described in the presented article is stored in the bryophyte herbarium of the Władysław Szafer Institute of Botany, Polish Academy of Sciences (KRAM). See Table 1 which lists species, collector, collection year, collection number, and collection site details. The sequences are available at GenBank: ITS: MN179594–MN179605 phy2 gene: MN239177 and MN239178 psbAF-trnHR2: MN239114–MN239130 atpI-atpH: MN239131–MN239140 trnL-trnF: MN239141–MN239153 rps4 gene: MN239154–MN239164 atpB1-rbcL1: MN239165–MN239176 psbB-clpP: MT173790–MT173792.
