# Supplementary material for: At the crossroads of botanical collections and molecular genetics laboratory: a preliminary study of obtaining amplifiable DNA from moss herbarium material
Source: PeerJ. 2020 May 26;8:e9109. doi: 10.7717/peerj.9109 (PMC7258893; doi:10.7717/peerj.9109)
Supplement: Supplemental Information 1 [file peerj-08-9109-s001.docx]

**a) Qiagen Kit extraction test**

| **No.** | **Species** | **Collector / Collection year** | **Collection number** | **Collection site details** |
| --- | --- | --- | --- | --- |
| **1** | *Andreaea depressinervis* Cardot | RO / 23.12.1979 | 4928/79 | **Maritime Antarctic, South Shetland Islands:**  **King George Island**  Admiralty Bay: mountain slope between Petrified Forest Creek and Ornithologist’s Creek, south of Arctowski Station called Ubocz.; lat. 62°10’S, long. 58°29’W; alt. 110 m a.s.l.; in dry, sheltered situation on gravelly ground among andesite rocks, forming loose cushions in *Usnea antarctica* community. |
| **2** | *Andreaea nitida* Hook.f. & Wilson | RO & CHB / 28.12.2006 | 3745/06 | **Subantarctica: Îles Kerguelen**  Grande Terre. Peninsule Courbet. Southern side of Val Studer: plateau at the south-eastern foot of Mont Crozier; lat. 49°17'47.9'' S, long. 70º02'35.6'' E; alt. 400 m a.s.l.; forming large monospecific patches on moist stone at stream bank in the stand of *Azorella selago*. |
| **3** | *Blindia magellanica Shimp.* | RO / 07.04.1999 | 611/99 | **Subantarctica, Prince Edward Islands:**  **Marion Island**  Mixed Pickle Cove on the west coast of the island, north-east of the hut towards the escarpment; lat. 46°52’16”S, long. 37°38’30”E; alt. 80 m a.s.l.; in *Blechnum penna-marina* fernbrakes, *Acaena magellanica* community, mires and springs. |
| **4** | *Brachythecium subplicatum* (Hampe) A.Jaeger | RO / 07.04.1999 | 614/99 | **Subantarctica, Prince Edward Islands:**  **Marion Island**  Mixed Pickle Cove on the west coast of the island, north-east of the hut towards the escarpment; lat. 46°52’16”S, long. 37°38’30”E; alt. 80 m a.s.l.; in *Blechnum penna-marina* fernbrakes, *Acaena magellanica* community, mires and springs. |
| **5** | *Breutelia integrifolia* (Taylor) A.Jaeger | RO & NP / 11.11.2006 | 124/06 | **Subantarctica, Îles Crozet: Île de la Possesion**  In the fellfield south-east of the Alfred Faure station toward the small stream; lat. 46°26'01.92''S, long. 51°51'43.34''E; alt. 100–120 m a.s.l.; in dry, deep fissures of black lava rocks in rocks. |
| **6** | *Bucklandiella heterostichoides* (Cardot) Bednarek-Ochyra & Ochyra | RO / 28.12.2006 | 3804/06 | **Subantarctica: Îles Kerguelen**  Grande Terre, Peninsule Courbet: a wide valley between Mont Amery and Mont Crozier; lat. 49º17'45.5''S, long. 70º01'36.5''E; alt. *ca* 450 m; in open places in fellfield on gravelly soil in dry situation, forming large monospecific patches. |
| **7** | *Cratoneuropsis chilensis* (Lorentz) Ochyra | RO / 08.04.1999 | 403/99 | **Subantarctica, Prince Edward Islands:**  **Marion Island**  Furseal Bay on the west coast of the island south of the hut; lat. 46°52’40”S, long. 37°37’57”E; alt. 25 m a.s.l.; in *Blechnum penna-marina* fernbrakes, mires, black lava rocks outcrops. |
| **8** | *Distichium capillaceum* (Hedw.) Bruch & Schimp. | RO / 03.12.2006 | 1198/06 | **Subantarctica: Îles Kerguelen**  Golfe du Morbihan. Presqu’ Ile Jeanne D’Arc: north-westernmost part of the peninsula, on the plateau and cliff overlooking the right-hand side of Ravin du Charpon, *ca* 1 km west of Port-Jeanne d’Arc; lat. 49º33'31.8'' S, long. 69º48’52.4’’E; alt. 153 m a.s.l.; on stony slope forming large pure tufts on damp soil in rock crevices on steep stream bank. |
| **9** | *Ditrichum strictum* (Hook.f. & Wilson) Hampe | RO / 11.11.2006 | 194/06 | **Subantarctica, Îles Crozet: Île de la Possesion**  eastern coast, plateau 2 km south of Port Alfred base and 2 km south-east of Mont Branca; lat. 46°44'59.6''S, long. 51°841'35.4''E; alt. 185 m a.s.l.; in crevices and on ledges of lava rock in dry and exposed situation associated with *Valdonia microcarpa*, *Bucklandiella membranacea* and *Andreaea acutifolia*. |
| **10** | *Hymenoloma antarcticum* (Müll. Hal.) Ochyra | RO / 12.03.1980 | 2665/80 | **Maritime Antarctic, South Shetland Islands:**  **King George Island**  Admiralty Bay, Martel Inlet, Precious Peaks; lat. 62º04’30’’S, long. 58º18’W; alt. 140 m a.s.l.; in crevices of andesite rocks on SW-facing slope. |
|  |  | JK / 15.02.1989 | Komárek sn. | **Maritime Antarctic, South Shetland Islands:**  **King George Island**  Fildes Peninsula: central mountains. |
| **11** | *Hymenoloma tortifolium* (Hook.f. & Wilson) Ochyra | RO / 21.12.2006 | 3344/06 | **Subantarctica: Îles Kerguelen**  Golfe du Morbihan. Ȋle Australia: the cliff overlooking north-western end of Lac Alicia and the first lake at its western foot; lat. 49º28'23.3''S, long. 69º52'30.0''E; alt. 55 m a.s.l.; on rocks at the foot of the cliff. |
| **12** | *Notoligotrichum trichodon* (Hook.f. & Wilson) G.L. Sm. | RO & MS / 25.11.1995 | 487/95 | **southern South America:**  **Isla Grande de Tierra del Fuego**  Argentina. Depto. Ushuaia: trail to Glaciar Martial above the upper chair-lift station; lat. 54^o^46’S, long. 68^o^29’W; alt. 900–1200 m a.s.l.; open forest dominated by *Nothofagus pumilio* with admixture of *N. betuloides* on ground by late snow patches. |
| **13** | *Polytrichadelphus magellanicus* (Hedw.) Mitt. | RO & MS / 21.11.1995 | 302/95 | **southern South America:**  **Isla Grande de Tierra del Fuego**  Argentina. Depto. Ushuaia: road to Lapataia, waterfalls of Rio Pipo; lat. 54^o^49’S, long. 68^o^30’W; *Nothofagus betuloides* and *N. pumilio* forest with dense thickets; on rocks on banks of the stream. |
| **14** | *Racomitrium lanuginosum* (Hedw.) Brid. | RO / 09.11.2006 | 15/06 | **Subantarctica, Îles Crozet: Île de la Possesion**  Eastern coast, Pointe Lieutard: rock outcrops 200 m south of Alfred Faure Station by the road toward the penguin rookery at Baie du Marin; lat. 46º26.125'S, long. 51º51.510'E; alt. 80 m a.s.l.; forming large extensive monospecific carpets on bare ground in the fellfield with *Azorella selago*, *Agrostis magellanica* and *Racomitrium lanuginosum* in dry and exposed situations, fertile. |
| **15** | *Sanionia uncinata* (Hedw.) Loeske | RO / 20.02.1980 | 2268/80 | **Maritime Antarctic, South Shetland Islands:**  **King George Island**  Admiralty Bay: Klekowski Crag between Lange Glacier and Polar Committee Glacier; lat. 62º07’30’’S, long. 58º30’W; alt. 250 m a.s.l.; in crevices of andesite rocks in dry situation. |
|  |  | RO / 09.11.2006 | 2/06 | **Subantarctica, Îles Crozet: Île de la Possesion**  eastern coast: Port Alfred Faure station over Baie du Marin, fellfield near the chapel toward the seashore; lat. 46º25'55.89''S, long. 51º51'36.68''E; alt. 120 m a.s.l.; in seepy place associated with *Agrostis magellanica* and *Breutelia integrifolia*. |
| **16** | *Sanionia georgicouncinata* (Müll. Hal.) Ochyra & Hedenäs | RO / 10.01.1980 | 454/80 | **Maritime Antarctic, South Shetland Islands:**  **King George Island**  Admiralty Bay, Keller Peninsula: British Point east of British Base; lat. 62º05’S, long. 58º23’30’’W; alt. 3 m a.s.l.; on raised marine beach forming extensive carpets in wet situation. |
| **17** | *Schistidium falcatum* (Hook.f. & Wilson) B. Bremer | RO / 09.01.1980 | 437/80 | **Maritime Antarctic, South Shetland Islands:**  **King George Island**  Admiralty Bay, Keller Peninsula, Tyrell Ridge: between Mt. Flagstaff and Piasecki Pass above the abandoned British Station; lat. 62º04’S, long. 58º24’30’’W; alt. 220 m a.s.l.; on bare earth on level ground in moist situation, associated with *Sanionia uncinata* and *Leptogium puberulum*. |
|  |  | RO & MS / 23.11.1995 | 408/95 | **southern South America:**  **Isla Grande de Tierra del Fuego**  Argentina. Depto. Ushuaia: trail to Glaciar Martial; lat. 54°46' S, long. 68°29’ W; alt. 600–1000 m a.s.l.; open forest dominated by *Nothofagus pumilio* with admixture of *N. betuloides*; on wet stone in stream bed. |
|  |  | RO & VRS / 21.04.1999 | 1447/99 | **Subantarctica, Prince Edward Islands:**  **Marion Island**  A small stream in the midway between the Kildalkey Bay Hut and Green Hill on the south-eastern coast; lat. 46°57’41”S, long. 37°51’06”E; alt. 70 m a.s.l.; *Blechnum penna-marina* fernbrakes, stream, stones. |
| **18** | *Schistidium halinae* Ochyra | RO / 16.03.1980 | 2711/80 | **Maritime Antarctic, South Shetland Islands:**  **King George Island**  Admiralty Bay: Puchalsi Peak between Nature Conservation Glacier and Rybak Glacier; lat. 62º10’S, long. 58º17’30’’W; alt. 140 m a.s.l.; on dry, bare andesite rocks on western slope. |
| **19** | *Schistidium* sp. Ochyra | RO with CHB, ML & DR / 08.12.2006 | 2022/06 | **Subantarctica: Îles Kerguelen**  Golfe du Morbihan. Anse de Saint Malo: in the middle of the eastern part of Île Guillou between Île Longue and Presqu’île de l’Amiral Douglas ; lat. 49º28'29.3''S, long. 69º48'37.7'' E; alt. 70–80 m a.s.l.; on moist stone. |
| **20** | *Valdonia microcarpa* (Mitt.) Ochyra | RO / 08.04.1999 | 555/99 | **Subantarctica, Prince Edward Islands:**  **Marion Island**  Kamskoppie south of Furseal Bay on the west coast of the island; lat. 46°53’25”S, long. 37°37’50”E; alt. 170 m a.s.l.; black lava rocks. |
| **21** | *Warnstorfia fontinaliopsis* (Müll. Hal.) Ochyra | RO / 27.01.1980 | 1193/80 | **Maritime Antarctic, South Shetland Islands:**  **King George Island**  Admiralty Bay: Blue Dyke at the entrance to bay on the west coast, south of Demay Point; lat. 62º13’40’’S, long. 58º27’W; alt. 40 m a.s.l.; in wet situation forming extensive mats and carpets in local depression, partly submerged. |

CHB – Ch. Brumpt (University of Rennes 1, Paimpont, France);

DR – D. Renault (University of Rennes 1, Paimpont, France);

JK – J. Komárek (Institute of Botany, Academy of Sciences of the Czech Republic, Třeboň, Czech Republic);

ML – M. Lebouvier (University of Rennes 1, Paimpont, France);

MS – M. M. Schiavone (College of Natural Sciences and Institute Miguel Lillo, National University of Tucumán, Tucumán, Argentina);

NP – N. van der Putten (Vrije Universiteit Amsterdam, Earth and Climate Cluster, Amsterdam, The Netherlands);

RO – R. Ochyra (W. Szafer, Institute of Botany, Polish Academy of Sciences, Cracow, Poland);

VRS – V.R. Smith (University of Stellenbosch, Department of Botany, Stellenbosch, South Africa).

**b) CTAB extraction test**

| **No.** | **Species** | **Collector / Collection year** | **Collection number** | **Collection site details** |
| --- | --- | --- | --- | --- |
| **1** | *Brachythecium rutabulum* (Hedw.) Shimp. | RO / 19.04.1999 | 1363/99 | **Subantarctica, Prince Edward Islands:**  **Marion Island**  Rockhopper Bay north-west of the Meteorological Station; lat. 46°52´25ʺS, long. 37°21´21ʺE; alt. 20-50 m a.s.l.; coastal rocks, *Blechnum penna-marina* fernbrakes, mire with *Agrostis magellanica*. |
| **2** | *Breutelia integrifolia* (Taylor) A.Jaeger | RO & CHB / 27.12.2006 | 3597/06 | **Subantarctica: Îles Kerguelen**  Grande Terre, Peninsule Courbet: plateau north of Ravin de Mica towards Rivière du Sud and les Mamelles; lat. 49º16'02.0'' S, long. 70º02'29.1'' E; alt. 337 m a.s.l.; forming extensive monospecific patches in open and exposed situation on dry blocks in the fellfield. |
| **3** | *Bucklandiella striatipila* (Cardot) Bednarek-Ochyra & Ochyra | RO & CHB / 28.12.2006 | 3758/06 | **Subantarctica: Îles Kerguelen**  Grande Terre, Peninsule Courbet: southern side of Val Studer, plateau at the south-eastern foot of Mont Crozier; lat. 49°17'47.9'' S, long. 70º02'35.6'' E; alt. 400 m a.s.l.; forming large monospecific patches on bare ground in the fellfield in the stand of *Azorella selago*. |
| **4** | *Cratoneuropsis chilensis* (Lorentz) Ochyra | RO / 07.04.1999 | 403/99 | **Subantarctica, Prince Edward Islands:**  **Marion Island**  Furseal Bay on the west coast of the island south of the hut; lat. 46°52’40”S, long. 37°37’57”E; alt. 25 m a.s.l.; in *Blechnum penna-marina* fernbrakes, mires, black lava rocks outcrops. |
|  |  | RO & VRS / 21.04.1999 | 1448/99 | **Subantarctica, Prince Edward Islands:**  **Marion Island**  A small stream in the midway between the Kildalkey Bay Hut and Green Hill on the south-eastern coast; lat. 46°57’41”S, long. 37°51’06”E; alt. 70 m a.s.l.; *Blechnum penna-marina* fernbrakes, stream, stones. |
| **5** | *Holodontium strictum* (Hook.f. & Wilson) Ochyra | RO & CHB / 27.12.2006 | 3581/06 | **Subantarctica: Îles Kerguelen**  Grande Terre, Peninsule Courbet: plateau north of Ravin de Mica towards Rivière du Sud and les Mamelles; lat. 49º16'02.0'' S, long. 70º02'29.1'' E; alt. 337 m a.s.l.; forming extensive monospecific patches in open and exposed situation on dry blocks in the fellfield. |
| **6** | *Rhacocarpus purpurascens* (Brid.) Paris | HBO & RO / 13.02.2013 | 613/13 | **Australia: Tasmania**  Cradle Mountain National Park: Dove Lake south of Craddle Valley, Dove Lake Circuit on the east side of the lake; alt. 955–970 m, lat. 41º39.013'S – 41º40.133'S, long. 145º57.694'E – 145º57.771'S. |
| **7** | *Valdonia microcarpa* (Mitt.) Ochyra | RO / 08.04.1999 | 555/99 | **Subantarctica, Prince Edward Islands:**  **Marion Island**  Kamskoppie south of Furseal Bay on the west coast of the island; lat. 46°53’25”S, long. 37°37’50”E; alt. 170 m a.s.l.; black lava rocks. |

CHB – Ch. Brumpt (University of Rennes 1, Paimpont, France);

HBO – H. Bednarek-Ochyra (W. Szafer, Institute of Botany, Polish Academy of Sciences, Cracow, Poland);

RO – R. Ochyra (W. Szafer, Institute of Botany, Polish Academy of Sciences, Cracow, Poland);

VRS – Ch. Brumpt (University of Rennes 1, Paimpont, France).
