# Supplementary material for: At the crossroads of botanical collections and molecular genetics laboratory: a preliminary study of obtaining amplifiable DNA from moss herbarium material
Source: PeerJ. 2020 May 26;8:e9109. doi: 10.7717/peerj.9109 (PMC7258893; doi:10.7717/peerj.9109)
Supplement: Supplemental Information 2 [file peerj-08-9109-s002.docx]

| **Locality** | **Coll.#** | ***5,8SR***  ***ITS-2*** | ***phy2***  ***gene*** | ***psbAF***  ***trnHR2*** | ***atpI***  ***atpH*** | ***trnL***  ***trnF*** | ***rps4***  ***gene*** | ***atpB1***  ***rbcL1*** | ***psbB***  ***clpP*** |
| --- | --- | --- | --- | --- | --- | --- | --- | --- | --- |
| ***Blindia magellanica***  Subantarctica,  Prince Edward Islands,  Marion Island | 611/99 | MN179594 | — | MN239130 | MN239140 | MN239141 | MN239154 | MN239165 | — |
| ***Brachythecium subplicatum***  Subantarctica,  Prince Edward Islands,  Marion Island | 614/99 | MN179595 | — | MN239129 | MN239139 | MN239142 | MN239155 | MN239166 | MT173792 |
| ***Breutelia integrifolia***  Subantarctica,  Îles Crozet,  Île de la Possesion | 124/06 | MN179596 | — | MN239128 | MN239138 | MN239143 | MN239156 | MN239167 | — |
| ***Bucklandiella heterostichoides***  Subantarctica,  Îles Kerguelen | 3804/06 | MN179597 | — | MN239127 | — | — | — | — | — |
| ***Cratoneuropsis chilensis***  Subantarctica,  Prince Edward Islands,  Marion Island | 403/99 | MN179598 | — | MN239126 | MN239137 | MN239144 | MN239157 | MN239168 | — |
| ***Distichium capillaceum***  Subantarctica,  Îles Kerguelen | 1198/06 | MN179599 | MN239177 | MN239125 | MN239136 | MN239145 | MN239158 | MN239169 | MT173790 |
| ***Ditrichum strictum***  Subantarctica,  Îles Crozet,  Île de la Possesion | 194/06 | MN179600 | MN239178 | MN239124 | MN239135 | MN239146 | MN239159 | MN239170 | — |
| ***Hymenoloma antarcticum***  Maritime Antarctic,  South Shetland Islands,  King George Island | 2662/80 | — | — | MN239122 | — | — | — | — | — |
| ***Hymenoloma antarcticum***  Maritime Antarctic,  South Shetland Islands,  King George Island | Komárek  s.n. | — | — | MN239123 | — | MN239147 | — | MN239171 | — |
| ***Racomitrium lanuginosum***  Subantarctica,  Îles Crozet,  Île de la Possesion | 15/06 | — | — | MN239121 | MN239134 | MN239148 | MN239160 | MN239172 | — |
| ***Sanionia uncinata***  Subantarctica,  Îles Crozet,  Île de la Possesion | 2/06 | MN179602 | — | MN239119 | MN239132 | MN239150 | MN239162 | MN239174 | MT173791 |
| ***Sanionia georgicouncinata***  Maritime Antarctic,  South Shetland Islands,  King George Island | 454/80 | MN179601 | — | MN239120 | MN239133 | MN239149 | MN239161 | MN239173 | — |
| ***Schistidium falcatum***  South America,  Isla Grande de Tierra del Fuego | 408/95 | MN179603 | — | MN239117 | — | — | — | — | — |
| ***Schistidium falcatum***  Maritime Antarctic,  South Shetland Islands,  King George Island | 437/80 | — | — | MN239116 | — | — | MN239163 | — | — |
| ***Schistidium falcatum***  Subantarctica,  Prince Edward Islands,  Marion Island | 1447/99 | — | — | MN239118 | — | MN239151 | — | — | — |
| ***Schistidium halinae***  Maritime Antarctic,  South Shetland Islands,  King George Island | 2711/80 | MN179604 | — | MN239115 | — | MN239152 | — | MN239175 | — |
| ***Warnstorfia fontinaliopsis***  Maritime Antarctic,  South Shetland Islands,  King George Island | 1193/80 | MN179605 | — | MN239114 | MN239131 | MN239153 | MN239164 | MN239176 | — |
