# Supplementary material for: At the crossroads of botanical collections and molecular genetics laboratory: a preliminary study of obtaining amplifiable DNA from moss herbarium material
Source: PeerJ. 2020 May 26;8:e9109. doi: 10.7717/peerj.9109 (PMC7258893; doi:10.7717/peerj.9109)
Supplement: Supplemental Information 3 [file peerj-08-9109-s003.pdf]

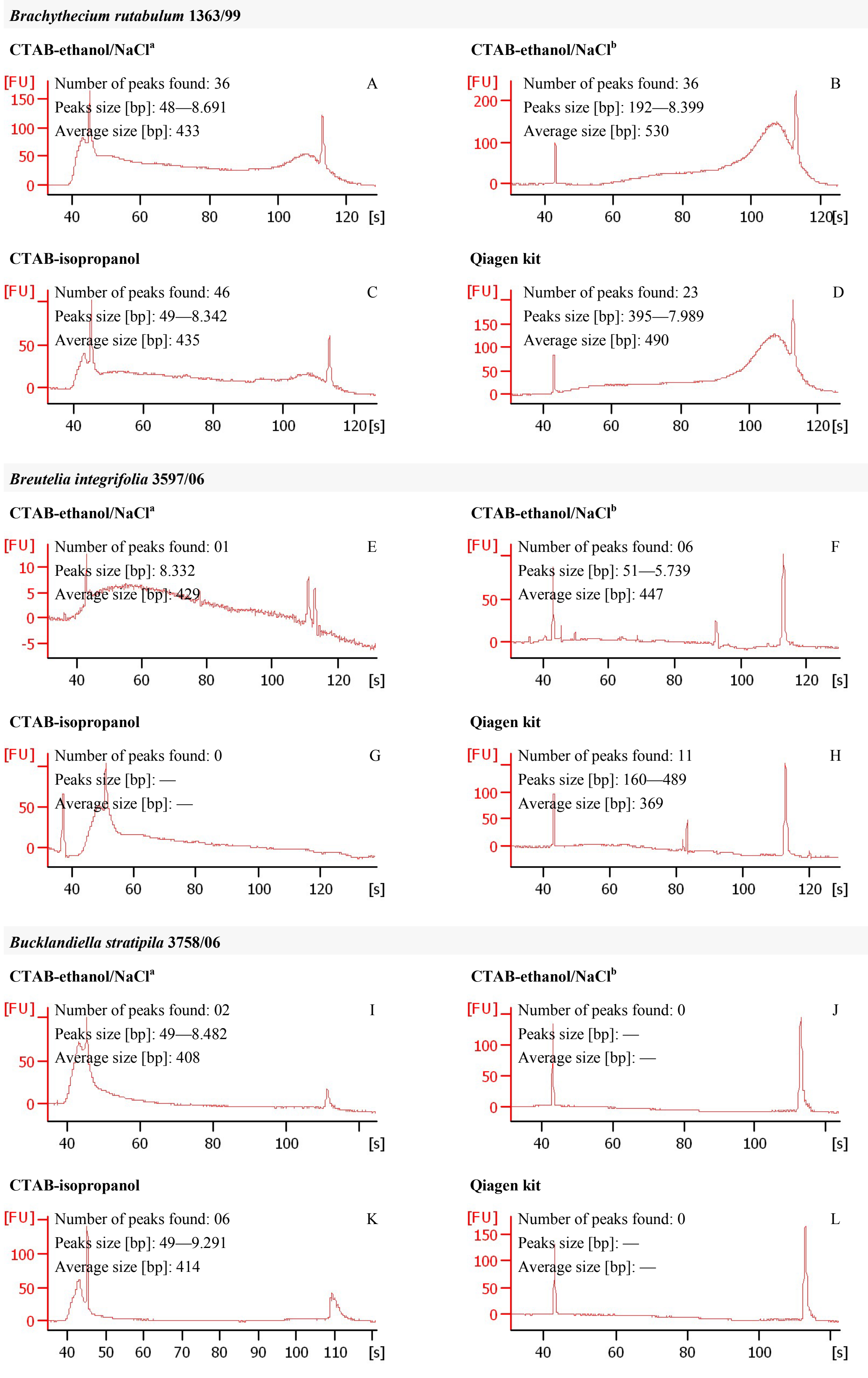

Brachythecium rutabulum 1363/99
CTAB-ethanol/NaCl[a]
Number of peaks found: 36
Peaks size [bp]: 48—8.691
Average size [bp]: 433
A
CTAB-ethanol/NaCl[b]
Number of peaks found: 36
Peaks size [bp]: 192—8.399
Average size [bp]: 530
B
CTAB-isopropanol
Number of peaks found: 46
Peaks size [bp]: 49—8.342
Average size [bp]: 435
C
Qiagen kit
Number of peaks found: 23
Peaks size [bp]: 395—7.989
Average size [bp]: 490
D
Breutelia integrifolia 3597/06
CTAB-ethanol/NaCl[a]
Number of peaks found: 01
Peaks size [bp]: 8.332
Average size [bp]: 429
E
CTAB-ethanol/NaCl[b]
Number of peaks found: 06
Peaks size [bp]: 51—5.739
Average size [bp]: 447
F
CTAB-isopropanol
Number of peaks found: 0
Peaks size [bp]: —
Average size [bp]: —
G
Qiagen kit
Number of peaks found: 11
Peaks size [bp]: 160—489
Average size [bp]: 369
H
Bucklandiella stratipila 3758/06
CTAB-ethanol/NaCl[a]
Number of peaks found: 02
Peaks size [bp]: 49—8.482
Average size [bp]: 408
I
CTAB-ethanol/NaCl[b]
Number of peaks found: 0
Peaks size [bp]: —
Average size [bp]: —
J
CTAB-isopropanol
Number of peaks found: 06
Peaks size [bp]: 49—9.291
Average size [bp]: 414
K
Qiagen kit
Number of peaks found: 0
Peaks size [bp]: —
Average size [bp]: —
L
[FU]
[s]

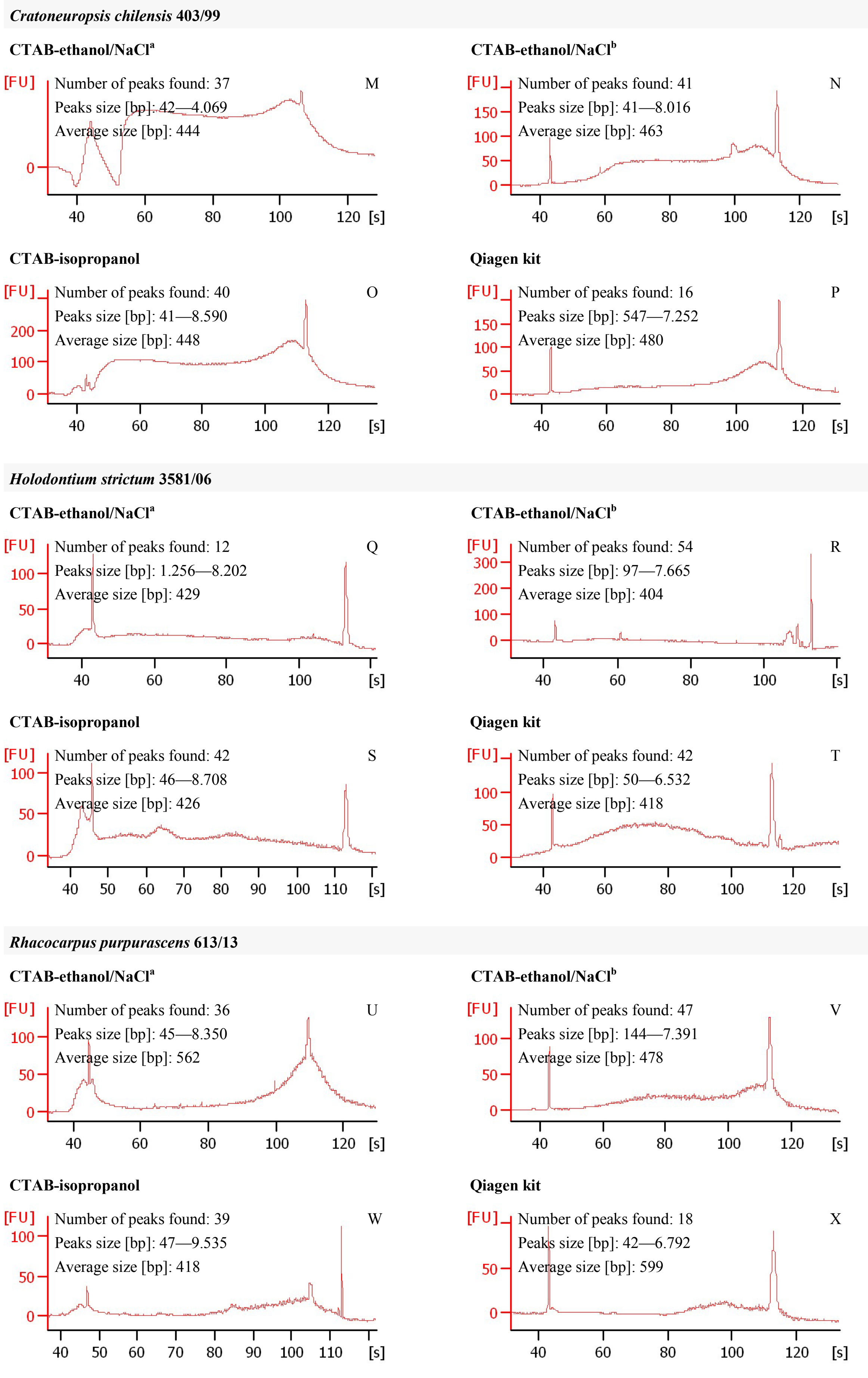
Cratoneuropsis chilensis 403/99
CTAB-ethanol/NaCl[a]
Number of peaks found: 37
Peaks size [bp]: 42—4.069
Average size [bp]: 444
M
CTAB-ethanol/NaCl[b]
Number of peaks found: 41
Peaks size [bp]: 41—8.016
Average size [bp]: 463
N
CTAB-isopropanol
Number of peaks found: 40
Peaks size [bp]: 41—8.590
Average size [bp]: 448
O
Qiagen kit
Number of peaks found: 16
Peaks size [bp]: 547—7.252
Average size [bp]: 480
P
Holodontium strictum 3581/06
CTAB-ethanol/NaCl[a]
Number of peaks found: 12
Peaks size [bp]: 1.256—8.202
Average size [bp]: 429
Q
CTAB-ethanol/NaCl[b]
Number of peaks found: 54
Peaks size [bp]: 97—7.665
Average size [bp]: 404
R
CTAB-isopropanol
Number of peaks found: 42
Peaks size [bp]: 46—8.708
Average size [bp]: 426
S
Qiagen kit
Number of peaks found: 42
Peaks size [bp]: 50—6.532
Average size [bp]: 418
T
Rhacocarpus purpurascens 613/13
CTAB-ethanol/NaCl[a]
Number of peaks found: 36
Peaks size [bp]: 45—8.350
Average size [bp]: 562
U
CTAB-ethanol/NaCl[b]
Number of peaks found: 47
Peaks size [bp]: 144—7.391
Average size [bp]: 478
V
CTAB-isopropanol
Number of peaks found: 39
Peaks size [bp]: 47—9.535
Average size [bp]: 418
W
Qiagen kit
Number of peaks found: 18
Peaks size [bp]: 42—6.792
Average size [bp]: 599
X
[FU]
[s]

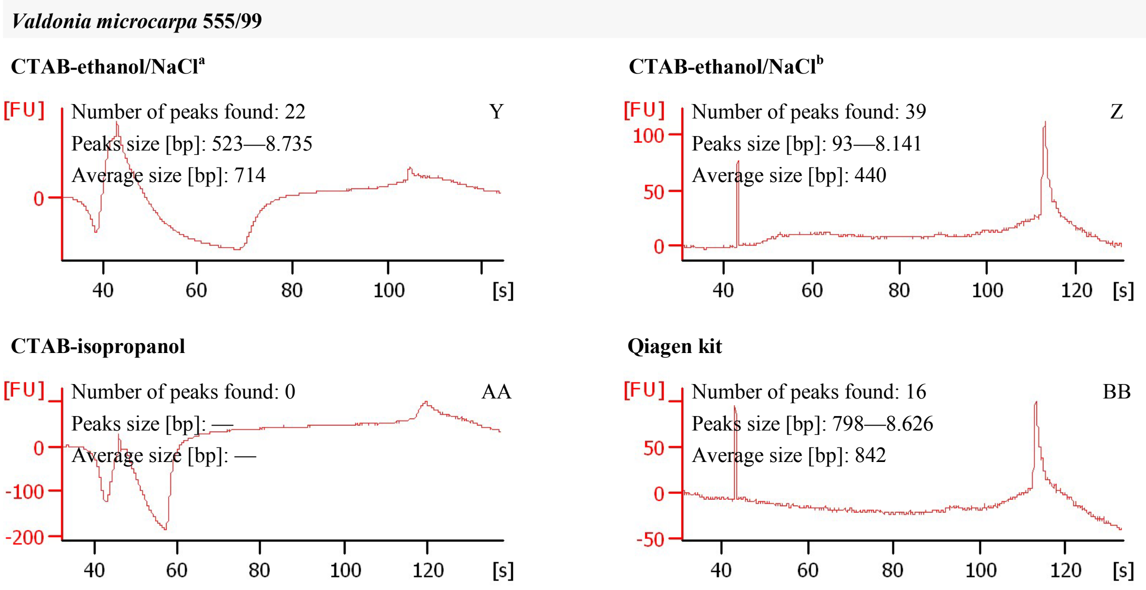
Valdonia microcarpa 555/99
CTAB-ethanol/NaCl[a]
[FU]
Number of peaks found: 22
Peaks size [bp]: 523—8.735
Average size [bp]: 714
Y
0
40
60
80
100
[s]
CTAB-ethanol/NaCl[b]
[FU]
Number of peaks found: 39
Peaks size [bp]: 93—8.141
Average size [bp]: 440
Z
100
50
0
40
60
80
100
120
[s]
CTAB-isopropanol
[FU]
Number of peaks found: 0
Peaks size [bp]: —
Average size [bp]: —
AA
0
-100
-200
40
60
80
100
120
[s]
Qiagen kit
[FU]
Number of peaks found: 16
Peaks size [bp]: 798—8.626
Average size [bp]: 842
BB
50
0
-50
40
60
80
100
120
[s]
